# *Mycobacterium avium* subsp. *paratuberculosis* Antigens Elicit a Strong IgG4 Response in Patients with Multiple Sclerosis and Exacerbate Experimental Autoimmune Encephalomyelitis

**DOI:** 10.3390/life13071437

**Published:** 2023-06-25

**Authors:** Davide Cossu, Yuji Tomizawa, Kazumasa Yokoyama, Tamami Sakanishi, Eiichi Momotani, Leonardo A. Sechi, Nobutaka Hattori

**Affiliations:** 1 Department of Neurology, Juntendo University, Tokyo 1138431, Japan; 2 Biomedical Research Core Facilities, Juntendo University, Tokyo 1138431, Japan; 3 Department of Biomedical Sciences, Sassari University, 07100 Sassari, Italy; 4 Tosei Center for Neurological Diseases, Shizuoka 4180026, Japan; 5 Division of Cell Biology, Juntendo University, Tokyo 1138431, Japan; 6 Comparative Medical Research Institute, Tsukuba 3050856, Japan; 7 SC Microbiology, AOU Sassari, 07100 Sassari, Italy; 8 Neurodegenerative Disorders Collaborative Laboratory, RIKEN Center for Brain Science, Saitama 3510918, Japan

**Keywords:** mycobacteria, multiple sclerosis, IgG4, experimental autoimmune encephalomyelitis, heath shock protein 70

## Abstract

Neuroinflammation can be triggered by microbial products disrupting immune regulation. In this study, we investigated the levels of IgG1, IgG2, IgG3, and IgG4 subclasses against the heat shock protein (HSP)70_533–545_ peptide and lipopentapeptide (MAP_Lp5) derived from *Mycobacterium avium* subsp. *paratuberculosis* (MAP) in the blood samples of Japanese and Italian individuals with relapsing remitting multiple sclerosis (MS). Additionally, we examined the impact of this peptide on MOG-induced experimental autoimmune encephalomyelitis (EAE). A total of 130 Japanese and 130 Italian subjects were retrospectively analyzed using the indirect ELISA method. Furthermore, a group of C57BL/6J mice received immunization with the MAP_HSP70_533–545_ peptide two weeks prior to the active induction of MOG_35–55_ EAE. The results revealed a significantly robust antibody response against MAP_HSP70_533–545_ in serum of both Japanese and Italian MS patients compared to their respective control groups. Moreover, heightened levels of serum IgG4 antibodies specific to MAP antigens were correlated with the severity of the disease. Additionally, EAE mice that were immunized with MAP_HSP70_533–545_ peptide exhibited more severe disease symptoms and increased reactivity of MOG_35–55_-specific T-cell compared to untreated mice. These findings provide evidence suggesting a potential link between MAP and the development or exacerbation of MS, particularly in a subgroup of MS patients with elevated serum IgG4 levels.

## 1. Introduction

While the precise pathological mechanism remains unclear, the influence of pathogen exposure as an environmental trigger for multiple sclerosis (MS), a chronic disease impacting the central nervous system (CNS), has been recognized. MS is characterized by inflammatory demyelination in the early phase of relapsing-remitting MS (RR-MS), followed by progressive phases dominated by neurodegenerative processes, resulting in the continuous loss of neurons and axons [1].

Although MS is not hereditary, genetic factors play a significant role in determining susceptibility to the disease, but they alone cannot fully explain its incidence [2]. The hygiene hypothesis proposes that early childhood exposure to pathogens may provide protective immunity, while infections during adulthood could act as triggers for autoimmunity, particularly in individuals with specific genetic predispositions [3]. Another possibility is the reactivation of viral or bacterial in individuals who had asymptomatic infections years earlier, potentially due to a weakened immune system or other external factors [4]. For instance, it has been suggested that the *Epstein–Barr* virus (EBV), which is considered the most influential risk factor for MS, could induce the expression of endogenous retroviruses from the *HERV-W* family, leading to the onset of MS [5]. Moreover, the variation in MS incidence prevalence across different geographical regions suggests that an abnormal immune response might be triggered by a region-specific pathogen prevalent in areas with high MS rates [1]. 

In the absence of a confirmed pathogen directly causing MS, the interplay between pathogens could have a significant impact on the pathogenesis of the disease. It is highly plausible that bacterial-viral coinfections could contribute to the disparity in MS risk between different regions. 

Among bacterial factors, exposure to antigenic determinants of *Mycobacterium avium* subsp. *paratuberculosis* (MAP), the etiological agent of paratuberculosis (commonly known as Johne’s disease) in animals, has been linked to the risk of developing MS [6,7,8,9]. Multiple clinical studies conducted in various countries consistently demonstrated an association between MAP and MS, based on the detection of mycobacterial DNA, as well as the presence of humoral and antigen-specific immune responses against MAP antigens in the sera and cerebrospinal fluids of patients with RR-MS [9]. It is important to note that the bacterium has never been isolated from any MS patient, suggesting that its role in MS, particularly in regions with low paratuberculosis prevalence, may be more related to the ingestion of antigenic components through contaminated food rather than active infection [10]. 

The potential of MAP antigens to exacerbate the progression of experimental autoimmune encephalomyelitis (EAE), a commonly used animal model of neuroinflammation, has been demonstrated. MAP can serve as an adjuvant, replacing *Mycobacterium tuberculosis*, to enhance EAE [11]. Additionally, it has been observed that oral administration of heath-killed mycobacteria activates mucosal immunity, modulates dendritic cells, and influences the trafficking of CD4 T-cells from mesenteric lymph nodes and spleen to the CNS, thereby worsening active EAE [12].

Furthermore, immune reactivity has been identified against cross-epitopes of MAP and EBV in individuals with MS, suggesting that both pathogens, through molecular mimicry, may activate a shared pathway leading to neuroinflammation in genetically susceptible individuals [13,14]. Studies have reported the presence of autoantibodies that recognize peptides from human myelin basic protein and interferon regulatory factor 5, which also cross-react with homologous peptides from EBV latent and lytic proteins, as well as MAP, in the sera and cerebrospinal fluid of MS patients [14].

In a recent study, specific antibodies against an epitope of the EBV nuclear antigen 1 (EBNA1) protein, specifically EBNA1_386–405_, which binds to the glial cell adhesion molecule (GlialCAM), exhibited increased immunoreactivity in both the serum and CSF of patients with RR-MS from the United States and German when compared to HCs [15]. 

In our previous study, we highlighted the high recognition of MAP_0106c_121–132_ and its homologous peptide EBNA1_400–413_, which shares a 5-amino acid overlap (GRRPF) with EBNA1_386–405_, in the serum and CSF of patients with RR-MS [14]. Furthermore, these peptides were found to induce both humoral and cell-mediated responses in RR-MS patients with a history of infectious mononucleosis [16]. 

Through the use of the Basic Local Alignment Search Tool (BLAST), we conducted an in silico analysis and discovered regions of local similarity between EBNA1_386–405_ and an epitope of MAP heath shock protein (HSP) 70, a protein that has previously been associated with MS [17].

Based on these findings, the objectives of our current study were as follows:

1. To evaluate the humoral response against the peptide MAP_HSP70_533–545_ in Japanese and Italian patients with RR-MS through in vitro experiments.
2. To characterize the clinical parameters associated with the IgG subclass response to MAP_HSP70_533–545_.
3. To assess the cross-reactivity between EBNA1_386–405_ and MAP_HSP70_533–545_ peptides.
4. To evaluate the impact of MAP_HSP70_533–545_ on neuroinflammation using an active EAE model.

## 2. Materials and Methods

### 2.1. Patients

A total of 130 serum samples were collected from individuals at Juntendo University School of Medicine, Tokyo, Japan, and another 130 serum samples were obtained from the University of Sassari, Sardinia, Italy. The study protocol received approval from the National Ethical Committee of the Juntendo University School of Medicine (Approval No. 205) and the Ethical Committee of the University of Sassari (Approval No. 2022/12524). All procedures involving human participants and animals were conducted in accordance with the approved guidelines. Prior to participating in the study, all subjects provided written informed consent. 

The diagnosis of RR-MS was made based on the McDonald criteria [18] for both Japanese (n = 65) and Italian (n = 65) patients. All patients included in this study had not received any treatment for at least 3 months prior to the sampling. The controls groups consisting of healthy individuals were selected to match the gender, ethnicity, and age distribution of the MS patient groups. Table 1 provides an overview of the baseline demographic and clinical characteristic of the subjects from Japan and Italy.

### 2.2. Antigens

Synthetic MAP_HSP70_533–545_ (CPPRRRSRAPPARR), EBNA1_386–405_ (CSQSSSSGSPPRRPPPGRRPF), and scramble (CRSSGPRPSRQGSPRSFPPSP) peptides were synthesized at >95% purity (ABclonal Biotechnology, Tokyo, Japan). MAP-specific lipopentapeptide MAP_Lp5 (DPhe-NMeVa1-Ile-Phe-Ala-OMe) was synthesized at >95% purity (GenScript, Piscataway, NJ, USA). Myelin oligodendrocyte glycoprotein (MOG)_35–55_ (MEVGWYRSPFSRVVHLYRNGK) peptide was synthesized at >95% purity (Synpeptide Co, Shanghai, China).

### 2.3. Peptide-Based Enzyme-Linked Immunosorbent Assays (ELISAs)

The perform the peptide-based indirect ELISA, we utilized the Imject maleimide-activated bovine serum albumin (BSA) spin kit (Thermo Fisher Scientific, Waltham, MA, USA). The kit was chosen to prevent the masking of antigenic epitopes, ensuring their accessibility for antibody binding. The procedure involved activating the BSA carrier protein with reactive sulfhydryl maleimide, purifying it, and then crosslinking it with the sulfidyl group (-SH) moiety in the cysteine-containing peptide antigen MAPHSP70_533–545_ following the manufacturer’s instructions. 

To determine the optimal coating conditions, titration experiments were conducted. Nunc-immuno-MicroWell-96-well solid plates (Thermo Fisher Scientific, Waltham, MA, USA) were coated with 50 µL/well of MAP_HSP70_533–545_ peptide diluted in ELISA coating buffer (Bio-Rad, Tokyo, Japan) at a final concentration of 10 μg/mL. The plates were incubated overnight at 4 °C. Subsequently, the microplate was blocked with 200 µL/well of Blocking One (Nakalai Tesque, Kyoto, Japan) for 1 h at room temperature. Sera samples were then added to the duplicate wells, diluted 1:100 in Blocking One, and incubated for 2 h at room temperature (25 °C). 

Following four washes with phosphate-buffered saline with 0.05% Tween 20 (PBS-T), the plates were incubated with 100 µL/well of horseradish peroxidase-labeled goat anti-human total IgG IgG1, IgG2, IgG3 or IgG4 antibodies (Southern Biotech Associates, Inc., Birmingham, AL, USA) for 1 h at room temperature. After incubation, the microplates were washed again, and the wells were incubated with 100 µL/well of ABTS Peroxidase System (SeraCare Life Sciences, KPL, Gaithersburg, MD, USA) for 10 min at room temperature in the dark. The optical density was measured at 650 nm using a Benchmark Plus Microplate Reader (Bio-Rad, Tokyo, Japan). Wells coated with BSA were included as a negative control, and the mean value obtained from these wells was subtracted from all other data points. The results were normalized against a positive control serum, which was included in all experiments.

### 2.4. Indirect ELISA for Anti-Lipopentapeptide (MAP_Lp5) Antibodies

In order to evaluate the specificity of our findings, all individuals who tested positive to MAP_HSP70_533–545_ were further tested for reactivity against the specific antigen MAP_Lp5. MAP_Lp5 is a distinctive antigenic lipoprotein found in the cell wall of MAP [19]. 

To eliminate any potential cross-reactivity with other mycobacterial components, all sera were pre-incubated with lyophilized *Mycobacterium phlei* obtained from the commercially available ELISA kit, Johnelisa II kit (Kyoritsu Seiyaku Corporation, Tokyo, Japan) [20]. This step ensured that the antibody response observed was specific to the antigens of interest. 

### 2.5. Inhibition ELISA

To determine the presence of cross-reactive antibodies between MAP_HSP70_533–545_ and EBNA1_386–405_ peptides in the sera of RR-MS patients, we conducted an inhibition ELISA. Serum samples were first pre-absorbed with saturating concentrations [10–15 mM] of EBNA1_386–405_, MAP_HSP70_533–545_, or scramble peptide overnight. This pre-absorption step aimed to block any cross-reactivity or binding of antibodies to these specific peptides.

After the incubation, the serum samples containing the antibody-antigen mixture were added to microplates coated with the MAP_HSP70_533–545_ peptide. The plates were then subjected to an indirect ELISA, following the previously described procedure. 

By pre-absorbing the serum samples with the respective peptides, any antibodies present in the samples that were specific to MAP_HSP70_533–545_ and EBNA1_386–405_ would already be bound to their corresponding peptide agent. As a result, the binding reaction in the wells of the ELISA microplate is reduced, and the reduction in absorbance in the wells is inversely proportional to the concentration of the analyte (cross-reactive antibodies) in the patient samples. 

### 2.6. Animal and Mouse Immunization

Groups of 9-week-old female wild-type C57BL/6J mice, with a total of 20 mice, were obtained from Charles River Laboratories, Yokohama, Japan, Inc. The mice were housed under pathogen-free conditions with a 12-h light/dark cycle. The animal experiments were conducted in accordance with the guidelines and approval of the Institutional Animal Care and Use Committee of Juntendo University School of Medicine (No. 290238). 

To induce EAE, mice were subcutaneously immunized with 200 μg of MAP_HSP70_533–545_ peptide emulsified in incomplete Freund’s adjuvant supplemented with 4 mg/mL of *Mycobacterium tuberculosis* H37Ra (CFA) two weeks prior to the induction of EAE. Placebo mice were immunized with a scrambled control peptide. 

For the induction of active EAE, mice were subcutaneously immunized with 200 μg of MOG_35–55_ peptide emulsified in CFA, followed by an intraperitoneal dose of 200 ng of pertussis toxin immediately after immunization and another dose 48 h later. The mice were monitored daily for clinical symptoms of EAE, and their disease severity was scored as follows: (1) for flaccid tail, (2) for impaired righting reflex and hind limb weakness, (3) for complete hind limb paralysis, (4) for complete hind limb paralysis with partial forelimb paralysis, and (5) for death. 

### 2.7. Histological Analysis and T-Cell Proliferation Assay

Histological analysis was performed on the spinal cord sections of EAE mice euthanized during the peak (14–16 days post-immunization) of clinical symptoms. The sections were stained with hematoxylin/eosin to detect inflammatory foci. The severity of inflammation was analyzed using a semiquantitative scale, where 1 represented a small infiltrate (<10 cells/field), 2 represented a medium infiltrate (>15 cells/field), and 3 represented a large infiltrate (>100 cells/field).

T-cell proliferation was assessed by measuring the incorporation of radioactive ^3^H-thymidine (1uCi/well) (PerkilnElmenr, Waltham, MA, USA) [21]. Spleen cells (4 × 10^5^ cells/well) from EAE mice at the peak of clinical symptoms were cultured for 2 days with 50 μg/mL MOG_35–55_ in the presence of gamma-irradiated (30.0 Gy) spleen cells (1 × 10^6^ cells/mL) syngeneic to the responding T-cells. DNA synthesis was determined by measuring the radioactivity of the incorporated-^3^H-thymidine with a scintillation plate counter (MicroBeta TriLux, PerkinElmer, Waltham, MA, USA) 18 h later. The proliferative response was expressed as a stimulation index (SI): counts per minute (cpm) of stimulated cells divided by counts per cpm of unstimulated cells.

### 2.8. Statistics

Statistical analysis was performed using Graphpad Prism 10 software (GraphPad Software, La Jolla, CA, USA). The non-parametric Mann–Whitney’s *u*-test was used to compare ELISA results between patients and HCs. Spearman’s correlation analysis was conducted to verify cross-reactivity between antibodies. Receiver operating characteristic (ROC) analysis was performed to assess the diagnostic accuracy of the ELISA and determine the cut-off for positivity with a specificity of 95%.

Clinical EAE scores were analyzed using the non-parametric Mann–Whitney *u*-test. Histological analysis of spinal cords was analyzed using one-way analysis of variance (ANOVA) followed by post-hoc Dunnett’s multiple comparison test. T-cell proliferation was analyzed using a two-tailed Student’s *t*-test. A *p*-value less than 0.05 was considered statistically significant.

## 3. Results

### 3.1. Anti-MAP_HSP70_533–545_ Antibodies Are Prevalent in Japanese and Italian RR-MS Patients

**Figure 1:**
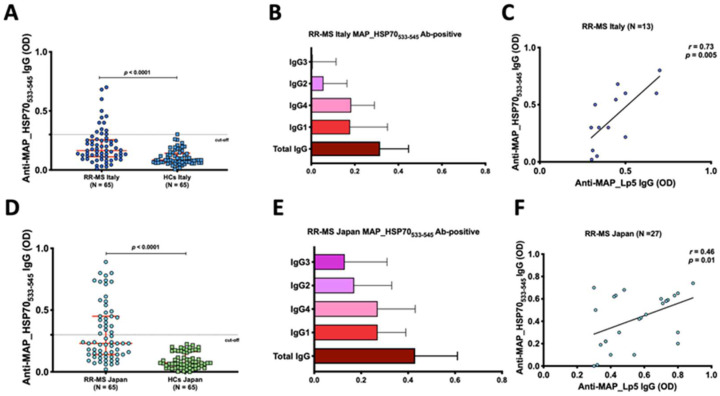
Evaluation of the humoral response to MAP_HSP70_533–545_ in Italian and Japanese populations. (**A**,**D**) Dot plots representing the distribution of total IgG detected by peptide-based ELISA. The red bars indicate the median values with interquartile range. The results are presented as the means of duplicate optical density (OD) values. (**B**,**E**) Characterization of IgG and subclasses in the sera of MAP_HSP70_533–545_ antibody positive patients. The bars represent the median values with interquartile range. (**C**,**F**) Correlation analysis between the titers of anti-lipopentapeptide (MAP_Lp5) antibodies detected by indirect ELISA with *Mycobacterim phlei* absorption, and the titers of anti-MAP_HSP70_533–545_ antibodies detected by peptide-based ELISA. Each circle on the graph corresponds to the titer of one serum sample. The Spearman’s correlation coefficient and statistical significance are indicated.

The peptide-based ELISA utilizing maleimide-activated BSA successfully detected total IgG and subclass levels (1–4). In the Italian cohort, using a cut-off of 0.4, a significant humoral response against MAP_HSP70_533–545_ peptide was observed in the serum of 13 out of 65 RR-MS patients (20%; 95% confidence interval [CI]: 23–46%), compared to 1 out of 65 (1.5%) HCs (*p* < 0.001) (Figure 1A). Among the IgG-positive RR-MS patients (n = 13), 6 (46%) exhibited high levels of IgG1, and 7 (54%) showed substantial levels IgG4, while IgG2 and IgG3 levels were not detected in the sera (Figure 1B). Additionally, there was a strong correlation between IgG4 levels and EDSS scores (*r* = 0.5, *p* < 0.0001). 

Similarly, in the Japanese RR-MS patients, at the same cut-off level, the MAP_HSP70_533–545_ peptide elicited strong antibody titers in the serum of 27 out of 65 (41%; 95% confidence interval [CI]: 23–46%), while no controls displayed positive results (*p* < 0.0001) (Figure 1D). IgG1 and IgG4 antibodies were elevated in the sera of 14 (52%) and 13 sera (48%), respectively (Figure 1E). A significant correlation between IgG4 and EDSS was also observed in Japanese RR-MS patients (*r* = 0.73, *p* < 0.0001). 

To confirm the specificity of the MAP_HSP70_533–545_ epitope, all subject sera were pre-adsorbed with *Mycobacterium phlei* antigen and tested against MAP_Lp5 antigen. Linear regression analysis demonstrated a good correlation between the ELISA based on MAP_HSP70_533–545_ and the MAP_Lp5- ELISA for both Italian (*r* = 0.73, *p* < 0.005) (Figure 1C) and Japanese (*r* = 0.46, *p* = 0.01) (Figure 1F) subjects, indicating the specificity of MAP detection. 

The indirect ELISA test showed good reproducibility, with an inter-assay coefficient of variation (CV) of 8% and an intra-assay CV of 4%.

### 3.2. Cross Reactivity between HSP70 and EBNA

**Figure 2:**
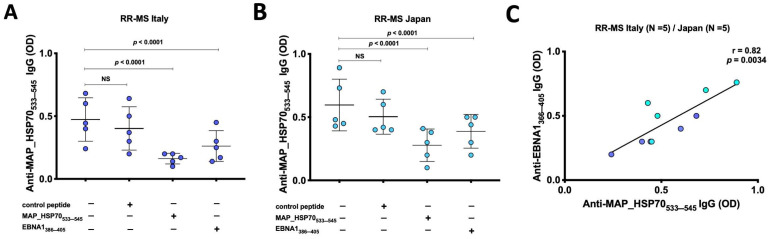
Inhibition ELISA assay. (**A**) Five sera from Italian RR-MS patients and (**B**) 5 sera from Japanese RR-MS patients were pre-incubated overnight with saturating concentrations of MAP_HSP70_533–545_, EBNA1_386–405_, or control peptide. After the incubation, the samples were added to plates coated with MAPHSP70_533–545_ peptide and subjected to indirect ELISA. The comparison of inhibition rates was calculated using Bland–Altman method. The results presented are representative of two separate experiments. (**C**) Correlation analysis between the titers of anti-EBNA1_366–345_ antibodies and the titers of anti-MAP_HSP70_533–545_ antibodies detected by peptide-based ELISA.

Inhibition immunoassays were conducted to investigate the presence of cross-reactive antibodies to MAP_HSP70_533–545_ and EBNA1_386–405_ peptides in the sera of RR-MS patients from Italy (Figure 2A) and Japan (Figure 2B). The results showed that EBNA1_386–405_ peptide inhibited the binding signal on MAP_HSP70_533–545_ coated plates by 35–47% *(p* < 0.0001). In comparison, the positive control (MAP_HSP70_533–545_ peptide) caused a decrease in the binding signal by 64–76%, while the negative control (scramble peptide) resulted in 15–17% reduction. Furthermore, a strong correlation was observed between the levels of anti-EBNA1_366–345_ antibodies and MAP_HSP70_533–545_ antibodies detected by peptide-based ELISA (*r* = 0.82, *p* < 0.0034) (Figure 2C). These findings not only support the association between EBV and MS but also suggest that MAP might be involved in the etiology or progression of the disease through a mechanism of molecular mimicry.

### 3.3. MAP_HSP70_533–545_ Immunization Exacerbates Active EAE

**Figure 3:**
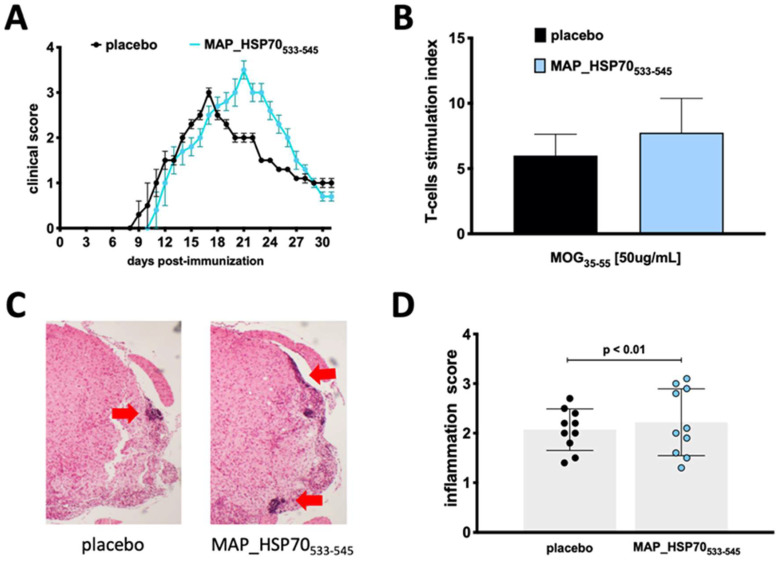
Effect of immunization with MAP_HSP70_533–545_ on active EAE. (**A**) Clinical score and course of the disease in C57BL/6J wild-type mice immunized subcutaneously with MAP_HSP70_533–545_ or placebo two weeks prior to EAE induction by MOG_35–55_. (**B**) T-cells isolated from the spleen were cultured with MOG_35–55_ peptide, the T-cell response was assessed by measuring [^3^H] thymidine incorporation at 48 h and analyzed at 56 h. (**C**) Spinal cord sections (4× magnification) were stained with hematoxylin/eosin during the peak of disease. (**D**) The inflammation index was calculated based on at least two sections per mouse. The data represent the combined results of three independent experiments with 10 mice per group. Data are expressed as mean ± standard deviation calculated using analysis of variance (ANOVA) followed by Dunnett’s post-hoc multiple comparison test.

To assess the encephalitogenic potential of MAP_HSP70_533–545_ in active EAE, groups of wild type mice (C57BL/6J) were immunized with the MAP_HSP70_533–545_ peptide two months prior to EAE induction. The immunized mice exhibited a slightly delayed onset of the disease (9 ± 0.4 placebo vs. 10 ± 0.5 immunized) but developed a more severe peak of the disease (3.0 ± 0.5 placebo vs. 3.5 ± 0.4 immunized) compared to non-immunized control mice (Figure 3A). The disease incidence (9/10 placebo vs. 9/10 immunized) and clinical course were comparable to placebo controls (Table 2). 

To determine whether MAP_HSP70_533–545_ affected the T-cell response to the MOG_35–55_ antigen, splenocytes from placebo-treated and MAP_HSP70_533–545_-immunized mice were isolated during the peak of clinical EAE and stimulated with MOG_35–55_ or a nonspecific stimulant, phytohemagglutinin (PHA). Proliferation was assessed using the ^3^H-thymidine incorporation assay. Splenocytes from MAP_HSP70_533–545_-immunized mice showed increased proliferation in response to MOG_35–55_ stimulation (Figure 3B). 

Furthermore, histological examination of spinal cords revealed an increased infiltration of mononuclear cells in MAP_HSP70_533–545_-immunized mice compared to placebo-treated mice (Figure 3C,D).

## 4. Discussion

In this study, we have provided further evidence for the involvement of mycobacteria in the pathology of MS by identifying a peptide derived from the MAP_HSP70 protein antigen that can elicit a strong immune response in patients with RR-MS. Additionally, we have demonstrated the role of this peptide in the neuroinflammation induced by the EAE model.

HSP70s are a group of highly conserved protein families that are induced under stress conditions, such as inflammation, and have been associated with neuroinflammation and neurodegeneration, particularly in RR-MS [22]. Through in silico analysis and the transcriptional profiling of MS patients compared to healthy controls, we observed a significant upregulation of several genes encoding HSP70s in various brain regions affected by MS, including the corpus callosum, hippocampus, internal capsule, and optic chiasm [22].

A separate study conducted on a cohort of 268 MS patients and 231 HCs from Sardinia demonstrated the presence of serum anti- MAP_HSP70 IgG in 23% of the patients, whereas only 6.5% of controls showed the presence of these antibodies [17]. 

BLASTp analysis revealed 28% amino acid identity between human HSP70 and MAP_HSP70, although no immunodominant epitope from the recombinant protein was identified.

Here, we have demonstrated the presence of cross-reactivity between MAP_HSP70_533–545_ and EBNA1_386–405_, which is a peptide involved in molecular mimicry with GlialCAM [15]. GlialCAM is an adhesion molecule primarily expressed in oligodendrocytes and astrocytes [23]. Mutations in the GlialCAM protein have been associated with megalencephalic leukodystrophy, a genetic neurodegenerative disorder that affecting the white matter of the CNS, which consists of glial cells and myelinated axons [24]. The shared linear amino acid sequence or structural similarities between MAP/EBV and GlialCAM epitopes may contribute to autoimmune processes.

When exposed to bacterial or viral antigens, the immune system undergoes adaptation, including changes in antibody affinity and isotype of peripheral B cells [25]. In patients with MS, the somatic hypermutation of B cells has been observed [26], potentially leading to the production of self-reactive antibodies that strongly interact with host proteins, such as GlialCAM. This interaction may trigger an immune response against GlialCAM-expressing-cells in the CNS, leading to inflammation and demyelination. 

In support of this hypothesis, we have detected IgG4 antibodies in serum of RR-MS patients (~50%) directed against MAP_HSP70_533–545_, particularly in patients with higher EDSS scores. Normally, IgG4 antibodies constitute around 5% of total IgG immunoglobulins [27]. The presence of IgG4 in RR-MS patients may be attributed to chronic exposure to MAP, primarily through the fecal-oral route. In Italy, paratuberculosis is endemic in domestic livestock in Sardinia [28], and several studies have demonstrated the presence of specific antibodies and MAP DNA in patients with autoimmune disorders, including MS, Chron’s diseases and type 1 diabetes [29]. As for the epidemiological situation of paratuberculosis in Japan, the disease’s prevalence is relatively low compared to Western countries [30]. However, seroprevalence studies have indicated the presence of IgG1 and IgG4 antibodies against MAP antigens in the serum of healthy individuals [20], as well as IgE in individuals with allergies [31], suggesting that the Japanese population may also be exposed to the mycobacterium, likely through the consumption of contaminated dairy products [20].

Furthermore, certain HLA DRB1*0405 alleles have been associated with an increased risk of developing MS in specific countries, such as Sardinia and Japan [32,33], as well as an increased susceptibility to IgG4-related disease in the Japanese populations [34].

Although the prevalence of MS is higher in Western countries compared to East Asia [35], one hypothesis is that individuals carrying this specific haplotype and exposed to MAP antigens, including MAP_HSP70_533–545_, may be more susceptible to developing autoimmune disorders. 

While there is limited literature on the effect of therapy on IgG4 in MS, a clinical study involving 29 Greek RR-MS patients demonstrated an increase in IgG4 levels after 24 months of treatment with alemtuzumab, a monoclonal antibody targeting CD52 [36]. Moreover, patients with higher IgG4 titers were more likely to develop secondary autoimmune disorders such as Crohn’s disease and thyroid-related disorders [36].

We have also demonstrated the direct impact of MAP_HSP70_533–545_ on the development of EAE. In C57Bl/6J mice, the exacerbation of EAE was likely attributed to autoreactive MAP_HSP70_533–545_ T-cells generated in response to immunization. Previous studies on EAE have revealed that various self-antigen specific T-cells contribute to autoimmune inflammation [37]. The delayed onset but more severe disease observed in mice may be associated with the chaperoning effect of HSP70 on regulatory T (Treg) cells’ function [38], potentially modulating the trafficking of Tregs between the periphery (where their accumulation may delay the onset of EAE) and the CNS (where a defective number is linked to disease severity). 

Interestingly, recent research has suggested that *Bacillus Calmette–Guérin* vaccination may reduce the incidence of MS through cytokine-induced IL-10 secreting CD8 T-cells, indicating that certain mycobacteria may have a role in disease prevention [21].

Although we have provided evidence supporting the association between MAP and the pathogenesis of MS, further investigation is needed to determine whether the antigenic components of MAP are recognized by T-cells and/or disease-causing autoreactive antibodies or if these antigens solely contribute to epitope spreading. To assess the encephalitogenic effect of MAP_HSP70_533–545_ T-cell during the effector phase of EAE, future experiments will involve inducing induce passive EAE through the adoptive transfer of these cells. 

Moreover, additional research will be conducted to comprehend the mechanism of infection-mediated neuroinflammation, with a specific focus on the role of mitochondria dysfunction. Mitochondria dysfunction has been associated with the development of both MS and EAE [39,40], as well as mycobacterial infection [41].

## Figures and Tables

**Table 1:** Demographic and clinical characteristics of Japanese and Italian cohorts.

	Japan		Italy	
	RR-MS (n = 65)	HCs (n = 65)	RR-MS (n = 65)	HCs (n = 65)
Gender female/male	45/20	40/25	46/19	47/18
Age mean ± SD	41 ± 14	43 ± 12	49 ± 9	45 ± 13
Age at onset mean ± SD	33 ± 10	0	38 ± 11	0
EDSS ^1^ score median (range)	2.5 (0–7)	0	3 (0–7.5)	0
Oligoclonal bands ≥2 (%)	57 (87)	0	44 (68)	0
IgG index ≥0.7 (%)	49 (75)	0	49 (70)	0
Albumin quotient ≥7 × 10^−3^ (%)	16 (25)	0	16 (24)	0

^1^ EDSS: Expanded Disability Status Scale.

**Table 2:** Effect of MAP_HSP70_533–545_ immunization on disease course of myelin oligodendrocyte glycoprotein (MOG)_35–55_-induced experimental autoimmune encephalomyelitis (EAE).

Treatment 2 Weeks before EAE Induction	Mouse Strain (Age)	EAE Incidence	Mortality	Mean Day of Onset	Peak Clinical Score
Placebo	C57BL/6 (9 weeks)	90%	0%	11 ± 0.5 *	3.0 ± 0.5
MAP_HSP70_533–545_	C57BL/6 (9 weeks)	90%	0%	10 ± 0.4	3.5 ± 0.4 *

* Statistically significant.

## Data Availability

The data underlying this article will be shared on reasonable request to the corresponding author.
